# Immunomodulation for the Treatment of Fungal Infections: Opportunities and Challenges

**DOI:** 10.3389/fcimb.2020.00469

**Published:** 2020-09-15

**Authors:** Muluneh Ademe

**Affiliations:** Department of Microbiology, Immunology and Parasitology, College of Health Sciences, Addis Ababa University, Addis Ababa, Ethiopia

**Keywords:** immunomodulation, fungal infections, antifungal therapy, cytokine therapy, cellular therapy

## Abstract

Opportunistic fungal infections are major causes of morbidity and mortality in patients with single or multiple defects in their immunity. Antifungal agents targeting the pathogen remain the treatment of choice for fungal infections. However, antifungal agents are toxic to the host mainly due to the close evolutionary similarity of fungi and humans. Moreover, antifungal therapy is ineffective in patients with immunosuppression. For this reason, there is an increased demand to develop novel strategies to enhance immune function and augment the existing antifungal drugs. In recent times, targeting the immune system to improve impaired host immune responses becomes a reasonable approach to improve the effectiveness of antifungal drugs. In this regard, immunomodulating therapeutic agents that turn up the immune response in the fight against fungal infections hold promise for enhancing the efficacy and safety of conventional antifungal therapy. In general, immunomodulating therapies are safe with decreased risk of resistance and broad spectrum of activity. In this review, therefore, clinical evidences supporting the opportunities and challenges of immunomodulation therapies in the treatment of invasive fungal infections are included.

## Introduction

Fungi cause a variety of clinical infections which range from asymptomatic-mild infections to potentially life-threatening systemic infections (Brown et al., 2012). Over the past few decades, the prevalence of fungal infections increased dramatically mainly due to the AIDS epidemics, irrational use of broad-spectrum antibiotics, the widespread use of intravascular catheters, advances in surgery and organ transplantation, and the increasing immune suppression (Segal et al., 2006; Rodloff et al., 2011). Each year, about 11.5 million life-threatening infections and more than 1.5 million deaths occur due to fungal infections (Bongomin et al., 2017).

Antifungal agents targeting the pathogen such as polyenes, azoles, flucytosine, and echinocandins are the treatment of choice for most fungal infections. However, the side effects associated with the use of antifungal agents, the appearance of resistant fungal strains, varied spectra of activity and failure to sterilize infected organs seriously limit the efficacy of antifungal chemotherapy (Neofytos et al., 2009). Particularly in severely immunosuppressed patients, response to systemic antifungal therapy alone remains disproportionately less satisfactory and cure may be improved if the immune deficit remits (Brown et al., 2012; Safdar, 2013). Currently, immunomodulating therapeutic agents that target the host immune response hold promise for improving conventional antifungal therapy (Casadevall and Pirofski, 2001). However, there is limited data concerning the benefit and clinical effectiveness of immunomodulation as an adjunct to antifungal therapy. Therefore, in this review, clinical evidences supporting the opportunities and challenges of immunomodulation therapies in the treatment of invasive fungal infections are discussed.

## Immunomodulating Therapies for Fungal Infections

The lower burden of fungal infections in people with intact immune response has been taken as strong evidence that normal immunity mediates effective resistance to fungal infections (Casadevall and Pirofski, 2001). In this regard, targeting the immune system to augment impaired host immune responses and thus enhance the efficacy of antifungal drugs becomes a reasonable approach to improve the prognosis of fungal infections (Casadevall and Pirofski, 2001; Segal et al., 2006). Immunomodulation, therefore, refers to a range of treatments aimed at harnessing patients' immune system to achieve control, stabilization, and potential eradication of disease (Sam et al., 2018). As mentioned below and summarized in Table 1, several immunomodulating approaches have been clinically tested for the treatment of fungal infections.

**Table 1:** Clinical benefits and challenges of immunomodulating agents used in patients with fungal infections.

**Immunomodulating Agents**	**Clinical Benefit**	**Challenges**
Adoptive T-cell therapy	Stimulation of T-cells with antigen and infusion into the patient (Tramsen et al., 2014)	Generation of an adequate number of fungal-specific T cells with sufficient purity is challenging (Papadopoulou et al., 2016)
Monoclonal antibodies	Avoid toxicity risks because they are directed specifically to pathogen epitopes, reduce durations of antifungal drug treatment (Casadevall et al., 2004; Cassone, 2008)	Highly specific, high production costs (Chames et al., 2009)
CAR T-cell therapies	T-cells are genetically modified to express CAR and provide MHC unrestricted antigen recognition (Meiliana et al., 2016)	Cytokine release syndrome and neurotoxicity (Kochenderfer et al., 2012; Brudno and Kochenderfer, 2016), longer time needed to generate autologous CAR T-cells (Neelapu et al., 2017), allogeneic T-cells may cause GvHD, and may be rapidly eliminated by the host immune system (Depil et al., 2020)
Dendritic Cells (DCs)	Leads to activation of specific T-cells and secretion of cytokines and chemokines. It can be used both in immunotherapy and vaccination (Lauruschkat et al., 2018)	Cost inefficient, difficult to scale, and labor intensive (Lauruschkat et al., 2018)
G-CSF	Restores neutrophil counts (Wright et al., 2017)	Lineage-specific (Costa, 1998), may stimulate leukemia (Rowe, 2000)
GM-CSF	Stimulates proliferation and differentiation of hematopoietic progenitor cells. Enhances antimicrobial function of mature neutrophils and monocytes against fungal targets (Safdar et al., 2013). It has wide range of applications (Shiomi and Usui, 2015)	Faster depletion, less stability and low targeting efficiency (Vanitha et al., 2017), may stimulate leukemia (Rowe, 2000)
Granulocyte Transfusion	Increases neutrophil counts, augments the host's defenses and reverses the increased susceptibility to infections (Hickey and Kubes, 2009)	Limited success due to low granulocyte counts and short lifespan of granulocytes (Estcourt et al., 2016; West et al., 2017)
IFN-⋎	Enhance Th1 response and augments the antifungal activity of macrophages and neutrophils (Stevens et al., 2006)	Potential to induce exacerbation of tissue inflammation, ischemia, and necrosis (Safdar et al., 2005), may worsen GvHD in allogeneic HSCT recipients (Wang et al., 2009)
Interleukins	Enhance Th1-mediated immunity (Winn et al., 2003; Akdis et al., 2011)	Unintended deleterious effects (Casadevall and Pirofski, 2001)
M-CSF	Promotes the growth of macrophages, increases phagocytosis, chemotaxis, and secondary cytokine production in monocytes and macrophages (Kandalla et al., 2016)	In cancer patients, it may worsen disease progression by enhancing macrophage population (Medina-Echeverz et al., 2014)
NK cell therapy	Release soluble factors (such as IFN-γ) which mediate antifungal activity. It may have activity against a wide spectrum of fungi (Schneider et al., 2016)	As shown in malignancies, evasion from NK cells control may limit the success of NK cell therapy (Davies et al., 2014)
Pathogen recognition receptors (TLR and PTX3)	Recognize motifs on fungal species and induce inflammatory responses. It is useful for TLR-defective individuals (Netea et al., 2006)	Difficult for manufacturing on a commercial scale, complex and unpredictable mode of action (Zeromski et al., 2019)
TNF-α	Stimulates PMNs (Lauruschkat et al., 2018)	Hepatotoxicity, nephrotoxicity, and neurotoxicity after systemic administration (Lauruschkat et al., 2018)

### Adoptive T-Cell Therapy

Adoptive T-cell therapy involves harvesting of T-lymphocytes from a patient or donor's blood, stimulating the cells to grow and expand in an *in vitro* system, and subsequently re-infusing the cells back into the patient. In this technique, high numbers of specific T-cells are injected into the patient where they recognize their target and aid the immune system in its elimination (Papadopoulou et al., 2016). Adoptive T-cell therapy is usually used after allogeneic stem cell transplantation (allo-SCT) because the adaptive immune system reconstitutes much slower than the innate immune system and artificial increase of specific T-cells helps to clear fungal infections (Bacher et al., 2015).

Adoptive therapy using *in vitro*-expanded fungus-specific T cells has shown clinical efficacy in murine and human clinical studies. As reviewed in Deo et al. the use of Apsergillus-specific CD4+ T cells isolated from the spleens of immunized mice which were re-stimulated *in vitro* were protective and extended the life of mice (Deo and Gottlieb, 2015). In a clinical trial by Perruccio et al. ten haploidentical stem cell transplant recipients with evidence of invasive aspergillosis received a single infusion of 1 × 10^5^-1 × 10^6^ cells per kg of expanded donor-derived anti-Aspergillus T-cell clones, and 9 of 10 patients cleared the infection within 7.8 ± 3.4 weeks (Perruccio et al., 2005).

Adoptive cell therapy is a promising tool for the fight against fungal infections. However, generating an adequate number of fungal-specific T-cells with sufficient purity as per the guidelines of Good Manufacturing Practice (GMP) is the major limitation (Papadopoulou et al., 2016). Moreover, certain immunosuppressants which are frequently used after allo-SCT such as cyclosporine A, methylprednisolone, and mycophenolic acid may interfere with adoptive T-cell transfer by lowering the number and activation of specific protective Th1 cells (Tramsen et al., 2014).

### Chimeric Antigen Receptor (CAR) T-Cell Therapy

CARs are artificially designed receptors that are introduced into T-cells. The CAR modification allows T-cells to execute their killing command without the need to bind to other receptors (Meiliana et al., 2016). CAR T-cell therapies were approved by the U.S. FDA for use in cancer (FDA news release, 2017), and the success of CAR T-cells in B cell malignancies led to the attempt to use CARs for infections including fungal diseases. Dectin-1, a naturally occurring receptor of the innate immune system that is not expressed on T-cells, has been targeted for CAR therapy. ß-glucan, the ligand for Dectin-1, is a polysaccharide found on the surface of many fungi (Lauruschkat et al., 2018). Kumaresan et al. constructed a CAR T-cell adapting the fungal receptor Dectin-1 for Aspergillus to activate T-cells via chimeric CD28 and CD3-ζ. In this study, the Dectin-CAR was activated by ß-glucan and the growth of *Aspergillus fumigatus* was inhibited (Kumaresan et al., 2014). CAR T-cells are one of the most promising immunotherapeutic tools and major histocompatibility complex (MHC) unrestricted antigen recognition is the main advantage of CAR T-Cell therapy. In recent times, repurposing T-cells through CAR T-cell therapy becomes an active area of research in the fight against infections and hematological malignancies. However, this therapeutic approach may provoke cytokine release syndrome (CRS) and neurotoxicity (Kochenderfer et al., 2012; Brudno and Kochenderfer, 2016). Moreover, the autologous generation of sufficient numbers of CAR T-cells may take several weeks, which might lose critical time in an acute infection like invasive fungal infections (Neelapu et al., 2017). Allogeneic CAR T-cell therapy, on the other hand, may give rise to off-the-shelf products with decreased cost and it can also be suitable for many patients as opposed to autologous CAR T-cell therapy in which each treatment must be made individually for each patient (Depil et al., 2020). Yet, allogeneic CAR T cells may cause life-threatening graft-vs.-host disease (GvHD), and these allogeneic T cells may also be rapidly eliminated by the host immune system, limiting their intended activity (Depil et al., 2020).

### Granulocyte Transfusion

Patients with leukemia or undergoing haematopoietic stem cell transplant (HSCT) are at higher risk of acquiring fungal infections (Grow et al., 2002). Prolonged neutropenia has become a major risk factor of invasive fungal infections and the spectrum of infections in neutropenic patients has shifted, with invasive fungal infections emerging as major determinants of morbidity and mortality (Marr et al., 2002). Without correction of neutropenia, antifungals alone may not resolve infections against which neutrophils form the primary line of defense. Therefore, in cases of drug-resistant fungal infections, granulocyte transfusion remains a logically attractive solution. Granulocytes engulf fungus, release antimicrobial peptides, and form extracellular traps (West et al., 2017). Granulocyte transfusion theoretically increases neutrophil counts, augments the host's defenses and reverses the increased susceptibility to infections (Hickey and Kubes, 2009).

The potential for leucocyte transfusion was established by early animal studies. In 1953, Brecher et al. showed that granulocytes transfused to neutropenic dogs migrated to areas of infection (Brecher et al., 1953). Since then, different studies supported the efficacy of granulocyte transfusion in invasive fungal infections. In a retrospective review by Diaz et al. 80% of children with granulocyte dysfunction or severe neutropenia who received granulocyte transfusion demonstrated response to invasive fungal infections (Díaz et al., 2014). Furthermore, among pediatric HSCT recipients treated with granulocyte transfusion, seven out of 14 patients with invasive fungal infections showed radiological improvement, with 79% 100-day survival (Nikolajeva et al., 2015). As reviewed by West et al. granulocyte transfusion together with granulocyte colony-stimulating factors (G-CSFs) yielded an overall response rate of 50–90% in invasive fungal diseases (West et al., 2017). However, the overall success of granulocyte transfusion is limited due to low granulocyte counts, low quality and short lifespan of the granulocytes (Estcourt et al., 2016; West et al., 2017).

### Dendritic Cells (DCs) Therapy

Dendritic cells recognize fungus by pattern recognition receptors and process fungal antigens. Activated dendritic cells secrete cytokines and chemokines, migrate to the lymph nodes and present antigens to specific T cells, which in turn are activated and primed (Bozza et al., 2003). In this approach, DCs can be stimulated with fungal antigens *ex vivo* and transfused to the patient. These DCs induce protective immune responses to the fungus due to activation of fungus-specific T cells (Roy and Klein, 2012). In a mouse model by Shao et al. DCs that had been transduced with IL-12 and stimulated by *A. fumigatus* were administered to neutropenic mice, and the DC therapy led to decreased mortality and fungal burden due to a strong Th1 response (Shao et al., 2005). Likewise, in a murine model, protective Th1 response was found when DCs were stimulated by *A. fumigatus* conidia and transfected with IL-12 (Bozza et al., 2003). However, despite the therapeutic potential of *ex vivo* DC stimulation in fungal infection, administration to the human patient is thought to be cost inefficient, difficult to scale, and labor intensive (Lauruschkat et al., 2018).

### Natural Killer (NK) Cell Therapy

NK cell therapy involves the transfer of NK cells from a donor to a patient. NK cells are the most significant contributor to IFN-γ secretion (Wang et al., 2012). Indeed, NK cell therapy is in its trial stage and data are scarce pertaining to its application in fungal infections. However, available reports showed that NK cell therapy has an immunotherapeutic potential against a wide spectrum of fungi, including *Aspergillus fumigatus, Cryptococcus neoformans, Rhizopus oryzae, Candida albicans, Paracoccidioides brasiliensis, and Mucorales* (Schneider et al., 2016). For instance, in allo-SCT patients, increased NK cell counts were associated with better control of invasive aspergillosis (Stuehler et al., 2015).

### Cytokine Therapy

Strengthening the immune system through administration of cytokines is the other approach to fight fungal infections. Cytokines modulate the immune response of the host by acting as signaling molecules that specifically induce the proliferation, differentiation, and activation or suppression of different target cells (Gulati et al., 2016). For instance, neutropenia predisposes cancer patients on corticosteroids to invasive fungal infections. Cytokines will shorten the duration of neutropenia by enhancing the phagocytic and killing activities of neutrophils, monocytes, and macrophages (Chiou et al., 2000; Winn et al., 2003). Cytokines used as immunomodulatory agents in fungal infection include colony-stimulating factors (CSFs), Interferon-Gamma (IFN-γ), TNF α and Interleukins.

### Interferon-Gamma (IFN-γ)

Interferon-gamma (IFN-γ) skews the immune response toward a protective Th1 phenotype. IFN-γ has been implicated as a treatment option in invasive fungal infections (Stevens et al., 2006). IFN-γ therapy was shown to augment the antifungal activity by significantly increasing the numbers of macrophages and neutrophils (Stevens et al., 2006; Lehrnbecher et al., 2011). In a randomized controlled trial on HIV positive patients with Cryptococcus meningitis, Jarvis and colleagues compared the addition of IFN-γ to standard amphotericin B therapy. In this study, Jarvis et al. showed that short-course IFN-γ therapy significantly increased the rate of CSF Cryptococcus clearance, with no significant increase in adverse events (Jarvis et al., 2012). Delsing et al. also recruited eight patients with invasive Candida and/or Aspergillus infections and administered IFN-γ together with standard antifungal therapy. In their finding, five of the eight patients treated with IFN-γ recovered from the invasive fungal disease (Delsing et al., 2014). Furthermore, three patients on renal transplant who were suffering from disseminated invasive aspergillosis were cured after 6 weeks of combined amphotericin B and IFN-γ treatment (Armstrong-James et al., 2010). A recent case report by Tsai and colleagues revealed that a child with life-threatening disseminated coccidioidomycosis had shown a reduced production of interferon-γ. The child was treated with interferon-γ together with antifungal therapy, and the treatment augmented type 1 immunity and resulted in complete resolution of the disease (Tsai et al., 2020). However, IFN-γ therapy in allogeneic hematopoietic stem cell transplant recipients may potentially worsen GvHD (Wang et al., 2009).

### Tumor Necrosis Factor α (TNF-α)

TNF-α stimulates polymorphonuclear neutrophils (PMNs), which in turn increase oxygen radical release and cause enhanced hyphal damage against fungal infections (Lauruschkat et al., 2018). In an earlier study by Nagai et al. administration of TNF-α to immunosuppressed mice in a model for pulmonary aspergillosis increased survival (Nagai et al., 1995). Moreover, in a mice model by Mehrad et al. intra-tracheal challenge with *A. fumigatus* conidia in both neutropenic and non-neutropenic BALB/c mice increased lung TNF-α levels. This also correlated with infiltration of mononuclear and polymorphonuclear cells (Mehrad et al., 1999). In the same study, neutralization of TNF-α caused an increase in lung fungal burden and mortality in both normal and neutropenic mice (Mehrad et al., 1999). However, toxicity following systemic administration, including hepatotoxicity, nephrotoxicity, and neurotoxicity are major limitations of TNF-α therapy (Lauruschkat et al., 2018).

### Interleukins

Interleukins are known to enhance Th1-mediated immunity which is essential for the protection against fungal pathogens (Akdis et al., 2011). For instance, IL-12 production is strongly correlated with the development of Th1 immunity through inhibition of Th2 type cellular responses which enhances host defense. In mice with neutropenia, IL-12 enhanced fluconazole efficacy against Candida infections. Moreover, IL-12 alone was shown to have an activity in experimental murine cryptococcosis, histoplasmosis, aspergillosis, and coccidioidomycosis (Mencacci et al., 2000; Winn et al., 2003). However, in human patients with autologous bone marrow transplants, 2 of 12 patients developed fatal fungal infections after IL-12 therapy. This event raised the concern that IL-12 administration may have unintended deleterious effects on immune function (Casadevall and Pirofski, 2001).

### Colony-Stimulating Factors (CSFs)

CSFs are secreted glycoproteins which bind to receptor proteins on the surfaces of hemopoietic stem cells. Through activation of intracellular signaling pathways, CSFs promote proliferation and differentiation of cells into a specific kind of blood cell (Sionov and Segal, 2003). CSFs are mostly used to accelerate myelopoiesis and augment phagocyte function. CSFs, including Macrophage CSF (M-CSF), G-CSF, and Granulocyte-Macrophage Colony-Stimulating Factor (GM-CSF) are used as immunomodulating agents for the treatment of fungal infections (Sionov and Segal, 2003; Sionov et al., 2005).

M-CSF promotes the growth of macrophages, increases phagocytosis, chemotaxis, and secondary cytokine production in monocytes and macrophages (Kandalla et al., 2016). M-CSF has been shown to be used for the treatment of fungal infections as adjunct therapy with other conventional antifungal agents. Kandalla et al. treated transplant-mouse models with M-CSF and found improved survival of the mice when challenged with *A. fumigatus*, from 10% in controls to 60% in M-CSF treated mice (Kandalla et al., 2016). On the other study by Hume and MacDonald, 46 stem cell transplantation patients with invasive fungal disease were given recombinant human M-CSF with conventional antifungal treatment, and patients who received M-CSF showed better survival as compared to historical controls (27 and 5%, respectively) (Hume and MacDonald, 2012). However, as tumor-associated macrophages represent up to 50% of the tumor cell mass in cancer patients, administration of M-CSF may accelerate disease progression by enhancing the macrophage population (Medina-Echeverz et al., 2014). In this regard, M-CSF is not usually recommended to be used in cancer patients with fungal infections.

G-CSF, on the other hand, promotes survival, proliferation, and differentiation of all cells in the neutrophil lineage. Plus, G-CSFs increase the function of mature neutrophils (Roberts, 2005). As chemotherapy may be myelo-suppressive causing neutropenia, G-CSF can be used adjunctly with conventional antifungal agents to restore neutrophil counts. In the study by Grigull et al. three children with proven fungal infections and hematological malignancies were treated with combination of G-CSF and antifungal therapy. The combination therapy effectively treated the fungal infection and all children survived both the underlying malignancy and the fungal infection (Grigull et al., 2006). Moreover, in HIV-infected patients with Aspergillus hyphae, G-CSF was shown to reverse neutrophil dysfunction (Wright et al., 2017).

While G-CSF is relatively lineage-specific, GM-CSF stimulates a wider range of immune cells (Costa, 1998). GM-CSF stimulates maturation of dendritic cells from monocyte precursors, differentiation of macrophages, and proliferation and activation of macrophages, monocytes, neutrophils, eosinophils, dendritic cells, and microglia (Shiomi and Usui, 2015). In this regard, GM-CSF has a theoretical advantage against wide range of fungal pathogens for which host defense is dependent on both neutrophil and macrophage function (Shiomi and Usui, 2015; Scriven et al., 2017). In a study by Giles et al. prophylaxis with GM-CSF for patients receiving chemotherapy to treat acute myelogenous leukemia led to a lower frequency of fatal fungal infections (1.9%) as compared to placebo (19%) (Giles, 1998). Chen et al. also examined the role of GM-CSF in patients with *Aspergillus ventriculitis* which has a high mortality (67%) with conventional treatment. In this study, GM-CSF was given as adjunct therapy in conjunction with voriconazole, amphotericin B, and caspofungin. After 2 years of therapy, patients fully recovered and remained in remission (Chen et al., 2017).

CSFs can be administered separately or in combinations with one or more CSFs. However, a better efficacy in reducing fungal disease incidence was reported when two or more CSFs are given combined with antifungal agents (Kuhara et al., 2000). For instance, in a clinical trial, Wan et al. compared the effect of prophylactic treatment of 206 allogenic HSCT recipients with G-CSF, GM-CSF, or a combination of both (G-CSF + GM-CSF). Their findings showed that invasive fungal disease related mortality after 600 days was lower in the groups who received G-CSF+GM-CSF (1.45%) or GM-CSF (1.47%) compared with G-CSF (11.59%) (*P* = 0.016) (Wan et al., 2015).

## Monoclonal Antibodies

The efficacy of therapeutic antibodies stems from various natural functions of antibodies including neutralization, antibody-dependent cell mediated cytotoxicity (ADCC,) and complement dependent cytotoxicity (CDC). Moreover, the antibody can be utilized as a drug delivery carrier (Suzuki et al., 2015). Humoral response is important for the host defense against fungal infections. Antibodies activate the classical pathway of the complement system and complement activation leads to the killing of fungi by neutrophils. In this regard, monoclonal antibodies (mAbs) can be used for immunotherapeutic purposes (Casadevall and Pirofski, 2012). Mycograb, for instance, is a human recombinant monoclonal antibody that was revealed to have synergy when combined with fluconazole, caspofungin, and amphotericin B against a broad spectrum of Candida species (Bugli et al., 2013). In a murine model, Matthews et al. tested the therapeutic potential of Mycograb with a combination of standard antifungal therapy, amphotericin B. Their findings showed a high overall response rate of Mycograb (84%) compared to controls (49%). The overall Candida-related mortality was also reduced from 18 (controls) to 4% (Mycograb) (Matthews et al., 2003). Rudkin et al. also generated a human recombinant anti- Candida mAbs (Rudkin et al., 2018). According to the report, the binding of mAb to *C. albicans* cell surface antigens promotes FcγR-dependent phagocytosis by macrophages, and it resulted in a reduced fungal burden in a murine model of disseminated candidiasis (Rudkin et al., 2018). Furthermore, a 4-year-old child with life-threatening disseminated coccidioidomycosis was observed to have an exaggerated production of interleukin-4. The child was treated with dupilumab, a monoclonal antibody that blocks the alpha chain common to the interleukin-4 and interleukin-13 receptors, and it resulted in rapid resolution of the clinical symptoms (Tsai et al., 2020). Moreover, as reviewed in Boniche et al. using monoclonal antibody-based immunomodulation therapies was shown to have a promising therapeutic potential over a wide range of fungal infections, including, *Histoplasma capsulatum, Aspergillus fumigatus, Pneumocystis jirovecii, Cryptococcus neoformans, Sporothrix schenckii*, and *Blastomyces dermatitidis* (Boniche et al., 2020). However, the majority of clinically utilized mAbs are chimeric, humanized or, fully human IgG1, produced by hybridoma technology, and the production of these mABs demands good manufacturing practice (GMP) conditions (Strohl, 2014; Rudkin et al., 2018). Moreover, the high production costs and high specificity restrain the extended use of mAbs (Chames et al., 2009).

### Toll-Like Receptors (TLRs)

Host immunity to recognize and respond to the fungal pathogen is mediated by a range of pathogen recognition receptors (PRRs) including TLRs (TLRs 2, 4, 7, 9). TLRs recognize motifs on fungal species and regulate the induced inflammatory responses (Plato et al., 2015). In view of this, TLR4 defective mice were shown to be more susceptible to *C. albicans* infection which is attributed to chemokine expression and neutrophil recruitment (Netea et al., 2006). These conceptions pave the way for the use of TLR agonists for the treatment of fungal infections which doesn't respond well in conventional antifungal treatments. In the study by Erbagci et al. an immunocompetent healthy patient who had lesions on her face for over 20 years caused by *Acremonium strictum* and which was unresponsive to topical and systemic antifungals was successfully treated with a TLR-7 agonist (topical imiquimod) (Erbagci et al., 2005).

### Pentraxin-3 (PTX3)

PTX3 is a pentraxin-related protein which is encoded by the PTX3 gene in humans. It is produced and released by different cells, including dendritic cells (DCs), mononuclear phagocytes, endothelial cells, and fibroblasts in response to primary inflammatory signals (Kunes et al., 2012). PTX3 enables pathogen recognition by macrophages and DCs through activation of the classical pathway of the complement system. PTX3 binds with high affinity to selected microorganisms, including *A. fumigatus*. In this regard, increased risk of fungal infections has been associated with deficiency of PTX3 (Daigo et al., 2016). In murine model, Pentraxin 3 deficient mice demonstrated defective recognition of conidia by alveolar macrophages and dendritic cells and they were highly susceptible to Aspergillus infection. Then again, administration of pentraxin 3 protected against Aspergillus challenge (Garlanda et al., 2002). Furthermore, in allogenic stem cell transplantation patients, the increased risk of invasive aspergillosis was linked to genetic deficiency of PTX3 (Cunha et al., 2014). While encouraging, the application of PRRs faces certain hurdles. On one hand, these natural products are usually polymeric, and unsuitable for manufacturing on a commercial scale. On the other hand, the mode of action is often complex and the effects in living organisms are unpredictable (Zeromski et al., 2019).

## Challenges of Immunomodulation Therapy

The number of anti-fungal resistant isolates is increasing and conventional antifungal drugs can have severe side effects to the host. Conversely, immunomodulating agents, in general, are reported to be safe with decreased risk of resistance and broad spectrum of activity (Lauruschkat et al., 2018; Sam et al., 2018). However, despite all the promising benefits, immunomodulation therapies used for treating fungal infections are still exploratory which involves complex as well as time-intensive genetic and cellular manipulations before use. Much more work has to be done to prove its efficacy in human trials (Lauruschkat et al., 2018).

Immunomodulation therapies are also cost-intensive, and the high costs may prohibit many patients from receiving this potentially life-saving therapy, especially patients located in the developing world where the burdens of fungal infections are expected to be high (Segal et al., 2006). Furthermore, immunomodulating therapies might sometimes be accompanied by severe side effects, such as toxicities, and inflammation. Pro-inflammatory cytokines, for instance, are essential to the host as mediators of inflammation and host resistance to infections. However, their overexpression leads to local and systemic toxicity (Netea et al., 2006). Likewise, administration of recombinant human G-CSF in *A. fumigatus* infected outbred mice antagonized the action of SCH56592 azole derivative, and resulted in large lung abscesses with increased fungal burden (Graybill et al., 1998).

## Conclusion

Immunomodulation approaches hold promise for improving the efficacy of antifungal therapy, subsequently decreasing the morbidity and mortality due to fungal infections. However, the use of immunomodulating agents to combat fungal infections is at the exploratory stage. To the date this review was done, most of imunomodulating agentstargeted for the treatment of fungal infections in humans are under clinical trials. However, considering the revolution of immunotherapies in treating cancer, immunomodulation may have the potential to become a game changer in the treatment of fungal infections, perhaps in the foreseeable future.

## Author Contributions

MA contributed to conception, design, acquisition, analysis and interpretation of data, and took part in drafting and revising the article. All authors contributed to the article and approved the submitted version.

## Conflict of Interest

The author declares that the research was conducted in the absence of any commercial or financial relationships that could be construed as a potential conflict of interest.

## Abbreviations

ADCC: Antibody-dependent cell mediated cytotoxicity
allo SCT: Allogeneic hematopoietic stem cell transplantation
CAR: Chimeric antigen receptor
CDC: complement dependent cytotoxicity
CSFs: Colony-stimulating factors
G-CSF: Granulocyte Colony-Stimulating Factor
GM-CSF: Granulocyte-Macrophage Colony-Stimulating Factor
M-CSF: Macrophage colony-stimulating factors
MHC: Major Histocompatibility Complex.
